# Aspirin 75 mg to prevent preeclampsia in high-risk pregnancies: a retrospective real-world study in China

**DOI:** 10.1186/s40001-023-01024-7

**Published:** 2023-02-02

**Authors:** Yue Xiao, Qi Ling, Mengxin Yao, Yingjie Gu, Yanshi Lan, Songliang Liu, Jieyun Yin, Qiuping Ma

**Affiliations:** 1 Taicang Affiliated Hospital of Soochow University, The First People’s Hospital of Taicang, 58 Changsheng Road, Suzhou, 215413 China; 2 Jiangsu Key Laboratory of Preventive and Translational Medicine for Geriatric Diseases, School of Public Health, Medical College of Soochow University, 199 Renai Road, Suzhou, 215123 Jiangsu China; 3 Department of Obstetrics and Gynecology, International Peace Maternity and Child Health Hospital, School of Medicine, Shanghai Jiao Tong University, Shanghai, China

**Keywords:** Real-world study, Preeclampsia, Low-dose aspirin

## Abstract

**Background:**

Several randomized clinical trials showed that aspirin could decrease the incidence of preeclampsia (PE) in women at high risk, but data from sources other than traditional clinical trials that investigating the preventive effect of aspirin 75 mg on PE is still lacking, especially in mainland China. We aimed to use Chinese real-world data to estimate the preventive effect of low-dose aspirin (LDA) on PE.

**Methods:**

Clinical data of pregnant women who were at high risk of PE and had their first prenatal visit at the affiliated Taicang People’s Hospital of Soochow University during November 31, 2018 and May 10, 2021 was retrospectively analyzed. Among the 266 included pregnant women, 115 individuals treated with aspirin 75 mg per day and the other 151 without such treatment were considered as the LDA group and the control group, respectively.

**Results:**

In the LDA group, 64 (55.65%) of 115 pregnant women took aspirin before 16 weeks of gestation. Besides, 12 (10.43%) and 34 (22.52%) women developed PE in the LDA group and control group, respectively; the aspirin prophylaxis was associated with a lower risk of PE (odds ratio = 0.40, 95% confidence interval = 0.20–0.82, *P* = 0.0098). In addition, LDA is slightly more effective when initiated before 16 weeks of gestation or in those without chronic hypertension, when compared with their counterparts.

**Conclusion:**

Prophylaxis with 75 mg per day of aspirin in high-risk women resulted in a significantly lower incidence of PE than that in the control group.

## Background

Preeclampsia (PE) is a multisystemic syndrome during pregnancy [1]. It is characterized by new-onset hypertension after 20 weeks of gestation, which progresses rapidly and can accompany organ dysfunctions of the maternal liver, kidney, brain, lung, and placental [2, 3]. Globally, PE affects 3–8% of pregnant women, and it is the second leading cause of maternal mortality as well as one of the leading causes of neonatal morbidity or mortality [4]. Meanwhile, its impact may persist even after pregnancy, as evidence suggested that affected mothers and their fetuses had an increased likelihood of cardiovascular diseases (CVD) later in life [5, 6]. Once it developed, termination of pregnancy remains the only effective treatment, which often leads to iatrogenic preterm birth [7]. Therefore, in an effort to improve adverse pregnancy outcomes, the current research efforts need to focus on not only the treatment, but also the early prevention of PE.

It is widely accepted that PE is derived from abnormal placentation with subsequent release of antiangiogenic markers, which evokes widespread inflammation, endothelial dysfunction and vasoconstriction, increasing placental platelet aggregation and thrombotic event [1, 7]. Aspirin, of which the main component is acetylsalicylic acid, has anti-inflammatory and anti-thrombotic properties, and is proposed as the most promising drug for the prevention of PE. Numerous large randomized controlled trials (RCTs), for example, the Aspirin for Evidence-Based Preeclampsia Prevention (ASPRE) [8] and ASPIRIN trial [9], have demonstrated that low-dose aspirin (LDA) started from the first trimester could significantly decrease the occurrence of PE, but without causing significant maternal or neonatal adverse outcomes [10, 11]. Therefore, as early as 2011, the World Health Organization (WHO) issued the bulletin suggesting that 75 mg of aspirin prophylaxis should be considered for individuals at high risk for PE [12]. Subsequently, the American College of Obstetricians and Gynecologists (ACOG) and the Society for Maternal–Fetal Medicine (SMFM) recommended aspirin prophylaxis (81 mg/d) for pregnancies at high risk of PE [13]. Additionally, the United States Preventive Services and Task Force (USPSTF) commissioned a systematic review to update the 2014 guideline [14], which determined that daily use of LDA reduced the risk of PE, preterm birth, fetal intrauterine growth restriction, and perinatal mortality [15]; also, this new version of recommendation was endorsed by ACOG and SMFM. In 2020, the Chinese Medical Association released updated clinical guidelines for the management of gestational hypertension and PE, recommending the prophylactic use of LDA (50 to 150 mg per day) in women who are considered to be at high risk for PE [16]. Meanwhile, other countries like the United Kingdom [17], Canada [18], and French [19] have also published similar recommendations.

Despite the beneficial of prophylactic aspirin for PE has been widely accepted, an appropriate dose of aspirin to prevent PE has not been established as different guidelines recommended dosages ranging from 60 to 150 mg. Interestingly, aspirin at doses of 60 mg [20, 21], 75 mg [22–24], 80 mg [25], 100 mg [26, 27], and 150 mg [28] have been shown to be effective in preventing PE in some studies, while contrary results were also reported (60 mg [29, 30], 75 mg [31], 80 mg [32, 33], 100 mg [34–36], and 150 mg [37]). This inconsistency may partly be explained by the varied methods for screening pregnancies at high risk of PE both within and across countries. In addition, although the Chinese Medical Association suggested the usage of LDA among high-risk pregnancies for PE [16], evidence of LDA to prevent PE in high-risk pregnancies is rare and inconsistent in mainland China. In 2020, a RCT in Shanghai of China analyzed the preventive effect of three-dose subgroups (25 mg, 50 mg, and 75 mg) of aspirin administration on the incidence of PE among high-risk pregnant women, which recommended 75 mg as a suitable dosage [23]. In contrast, a recent multicenter RCT, which has the largest sample size in this field in China to date, reported that 100 mg of aspirin per day did not significantly reduce the incidence of PE [36]. What is more, RCTs are usually performed under strict inclusion and exclusion criteria, so their representativeness and external validity are somewhat limited, whereas real-world studies use broader inclusion criteria and fewer exclusion criteria to obtain a set of subjects consistent with the extrapolated population of trial results, which greatly reduces selective bias. All the above suggest the necessity for providing local evidence, especially from real-world clinical practice.

Therefore, we aimed to perform a retrospective real-world study to investigate the preventive effect of LDA on pregnant women at high risk for PE in Taicang, China.

## Methods

### Study population

The current study was conducted at Taicang Affiliated Hospital of Soochow University, the First People's Hospital of Taicang, Suzhou, Jiangsu Province in eastern China. In 2020, obstetricians in the hospital started to routinely use LDA to prevent PE among high-risk pregnant women, who were defined as containing ≥ 1 high-risk factor or ≥ 2 intermediate-risk factors for PE according to the 2019 ACOG guidelines [38]. Briefly, a history of PE, multiple pregnancies, chronic hypertension, type 1 or type 2 diabetes, renal disease, and autoimmune diseases were listed as high-risk factors, while nulliparity, obesity [body mass index (BMI) ≥ 30 kg/m^2^], family history of PE (mother or sisters), sociodemographic characteristics (low socioeconomic status and African descent), advanced age (≥ 35 years), and personal medical history factors (history of low birth weight or small for gestational age at delivery, previous adverse pregnancy outcomes, and more than 10 years from previous pregnancy) were listed as intermediate-risk factors. Thereafter, eligible high-risk women were prescribed to take 75 mg of aspirin daily at bedtime from 12 to 24 weeks of gestation to 36 weeks of gestation or the occurrence of PE. Until May 10, 2021, 115 high-risk individuals who meet the above requirement were considered as the LDA group. Subsequently, we systematically searched the medical system and extracted information of pregnant women who had their first prenatal visit to the same hospital before their 24 gestational weeks from November 31, 2018 to January 19, 2020 when LDA was not implemented. As a result, 151 high-risk individuals, according to the same criteria as the LDA group, who did not take LDA were considered as the control group.

All participants provided written informed consent, and the study was approved by the ethics committee of the Taicang Affiliated Hospital of Soochow University.

### Data collection

At their first antenatal examination, demographic and clinical information of pregnant women was collected, including maternal age, pre-pregnancy BMI, obstetrical history, previous medical history (such as chronic hypertension, diabetes mellitus, PE, and family history of PE), gestational age and systolic blood pressure (SBP)/diastolic blood pressure (DBP). Additionally, the duration and dosage of aspirin prophylaxis were recorded for those taking LDA at subsequent antenatal examinations. In addition, D-dimer, platelet count, platelet aggregation rate, fibrinogen, prothrombin time, and systolic/diastolic (S/D) value of uterine artery were measured and recorded in the third trimester. Information on pregnancy complications, gestational age at delivery, mode of delivery, postpartum hemorrhage, and 1-min and 5-min Apgar scores after delivery, were also extracted from the medical records.

### Outcomes

The primary outcome is PE, defined as new-onset gestational hypertension (SBP ≥ 140 mmHg and/or DBP ≥ 90 mmHg) after 20 weeks of gestation with either proteinuria (≥ 300 mg/24 h or a protein/creatinine ≥ 0.3 or > 2 + by dipstick on 2 or more occasions) or features of end-organ dysfunction [3]. According to the gestational week of onset, PE is divided into late-onset (≥ 34 gestational weeks) and early-onset (< 34 gestational weeks). Secondary outcomes are gestational hypertension, cesarean section, postpartum hemorrhage (blood loss ≥ 500 ml for spontaneous delivery or ≥ 1000 ml for cesarean section), preterm birth (delivery before 37 weeks of gestation) [39], fetal intrauterine growth restriction, fetal distress, and neonatal intensive care unit (NICU) admission.

### Statistical analyses

The main characteristics of women between the LDA group and control group were compared using t-test, *χ*^*2*^ tests, Fisher’s exact tests or the rank sum tests, when appropriate. The logistic regression model was used to estimate the risk of PE by comparing the LDA group to the control group, expressed with odds ratios (OR) and 95% confidence intervals (CI). In addition, subgroup analyses according to the initiation time of LDA treatment and other ACOG risk factors were applied. A *P*-value for interaction was calculated to evaluate the differences in associations between LDA and different risk factors.

A two-sided *P*-value < 0.05 was considered statistically significant. Statistical Analysis System software (version 9.4, SAS Institute, Cary, NC, USA) was used to perform database management and statistical analysis.

## Results

A total of 266 pregnant women were eligible for the present study, including 151 being classified as the control group and 115 as the LDA group (64 initiated before 16 weeks of gestation), and women in the LDA group claimed that they took LDA as prescribed. Table 1 shows the characteristics of the study participants between the LDA group and the control group. The mean age of pregnant women was 32.21 ± 5.39 years, the mean gestational age was 37.48 ± 1.83 weeks, and the average pre-conceptional BMI was 24.89 ± 4.54 kg/m^2^. In general, demographic and clinical indicators measured in the third trimester were comparable between these two groups.

**Table 1:** Characteristics of the study population

Variables, n (%) or mean (SD)	LDA (*n* = 115)	Control (*n* = 151)	*P*
Maternal age (years)	31.99 (5.31)	32.38 (5.47)	0.5639
Delivery gestational age (week)	37.57 (1.73)	37.40 (1.91)	0.4543
Pre-pregnancy BMI (kg/m^2^)	25.81 (4.57)	24.19 (4.41)	0.0038
High risk factors			0.0560
History of PE	3 (2.61%)	7 (4.64%)	0.5220
Multiple pregnancy	24 (20.87%)	33 (21.85%)	0.8463
Chronic hypertension	7 (6.09%)	3 (1.99%)	0.1065
Pre-existing diabetes	11 (9.57%)	8 (5.30%)	0.1807
Kidney disease	0 (0.00%)	3 (1.99%)	0.2610
Autoimmune diseases	1 (0.87%)	0 (0.00%)	0.4323
Intermediate risk factors			0.1882
Nulliparous	56 (48.70%)	77 (50.99%)	0.7104
Obese (BMI ≥ 30 kg/m^2^)	26 (22.61%)	30 (19.87%)	0.5870
Family history of PE	4 (3.48%)	6 (3.97%)	0.8334
Age (≥ 35 years)	42 (36.52%)	72 (47.68%)	0.0684
Clinical indicators			
D-dimer (μg/L)	2273.90 (1212.50)	2476.60 (1827.30)	0.2790
Platelet count (10^9^/L)	197.00 (62.92)	195.80 (56.86)	0.8735
Platelet aggregation rate (%)	70.30 (5.37)	67.89 (4.38)	0.0017
Fibrinogen (g/L)	4.01 (0.82)	3.62 (0.69)	< 0.0001
Prothrombin time (s)	11.35 (0.89)	11.13 (0.87)	0.0504
S/D value of uterine artery	2.31 (0.35)	2.29 (0.35)	0.5406

*LDA* low-dose aspirin, *BMI* body mass index, *PE* preeclampsia, *S/D* systolic/diastolic

The incidence of PE was 10.43% in the LDA group compared to 22.52% in the control group, resulting in OR of 0.40 (95%CI = 0.20–0.82) (Table 2). The median (interquartile range) of gestational age at the onset of PE was 36 (34–38) weeks, and 84.78% (39/46) of patients suffered late-onset PE (≥ 34 gestational weeks). In addition, adverse maternal pregnancy outcomes (i.e., gestational hypertension, gestational diabetes, cesarean section, and postpartum hemorrhage) and fetal outcomes (i.e., preterm birth, NICU admission, intrauterine growth restriction, and fetal distress) were not significantly different between the LDA group and control group.

**Table 2:** Pregnant outcomes between the LDA and the control groups

Outcomes, (n%) or mean (SD) or median (interquartile range)	LDA (*n* = 115)	Control (*n* = 151)	OR (95%CI)	*P*
Primary outcome				
Preeclampsia	12 (10.43%)	34 (22.52%)	**0.40 (0.20–0.82)**	**0.0098**
Time of onset	37 (34.5–38)	35 (34–38)	–	0.5613
Early-onset (< 34 gestational weeks)	2 (1.73%)	5 (3.31%)	0.52 (0.10–2.71)	0.2323
Late-onset (≥ 34 gestational weeks)	10 (8.70%)	29 (19.21%)	**0.38 (0.18–0.82)**	**0.0116**
Secondary outcomes				
Gestation hypertension	19 (16.52%)	19 (12.58%)	1.38 (0.69–2.74)	0.3631
Gestational diabetes	36 (31.30%)	40 (26.49%)	1.27 (0.74–2.16)	0.3892
Cesarean section	87 (75.65%)	102 (67.55%)	1.49 (0.87–2.58)	0.1489
Postpartum hemorrhage	5 (4.35%)	4 (2.65%)	1.67 (0.44–6.37)	0.5066
Preterm delivery	25 (21.74%)	38 (25.17%)	0.83 (0.46–1.47)	0.5149
NICU admission	43 (37.39%)	56 (37.09%)	1.01 (0.61–1.67)	0.9593
Fetal intrauterine growth restriction	7 (6.09%)	14 (9.27%)	0.63 (0.25–1.63)	0.3400
Fetal distress	4 (3.48%)	6 (3.97%)	0.87 (0.24–3.16)	0.8334
1-min Apgar score	9.85 (0.48)	9.76 (0.81)	–	0.2719
5-min Apgar score	9.93 (0.41)	9.87 (0.68)	–	0.4031

*LDA* low-dose aspirin, *OR* (95%CI), odds ratio (95% confidence interval), *NICU* neonatal intensive care unit.

*P*-values <0.05 are emphasized in bold font.

In the LDA group, we found that women who took LDA before 16 weeks of gestation had a significantly lower incidence of PE than those after 16 weeks (4.69% vs.17.65%;* P* = 0.0239). Meanwhile, it was shown that chronic hypertension was related to a higher PE occurrence compared with those without chronic hypertension despite taking LDA (*P* < 0.0001, Table 3).

**Table 3:** PE occurrence in the LDA group

	Total	PE (*n* = 12)	non-PE (*n* = 103)	*P*
Onset of LDA				**0.0239**
≤ 16 gestational weeks	64	3 (4.69%)	61 (95.31%)	
> 16 gestational weeks	51	9 (17.65%)	42 (82.35%)	
ACOG Risk factor				
Age (years)				0.3811
≥ 35	42	3 (7.14%)	39 (92.86%)	
< 35	73	9 (12.33%)	64 (87.67%)	
BMI (kg/m^2^)				0.4640
≥ 30	26	4 (15.38%)	22 (84.62%)	
< 30	89	8 (8.99%)	81 (91.01%)	
History of PE				0.7163
Yes	3	0 (0.00%)	3 (100.00%)	
No	112	12 (10.71%)	100 (89.29%)	
Family history of PE				0.6395
Yes	4	0 (0.00%)	4 (100.00%)	
No	111	12 (10.81%)	99 (89.19%)	
Pre-existing diabetes				0.2328
Y es	11	2 (18.18%)	9 (81.82%)	
No	104	10 (9.62%)	94 (90.38%)	
Chronic hypertension				** < 0.0001**
Yes	7	4 (57.14%)	3 (42.86%)	
No	108	8 (7.41%)	100 (92.59%)	
Nulliparous				0.4803
Yes	56	7 (12.50%)	49 (87.50%)	
No	59	5 (8.47%)	54 (91.53%)	
Multiple pregnancy				0.7123
Yes	24	3 (12.50%)	21 (87.50%)	
No	91	9 (9.89%)	82 (90.11%)	

*PE* preeclampsia, *LDA* low-dose aspirin, *ACOG* American college of obstetricians and gynecologists, *BMI* body mass index.

*P*-values <0.05 are emphasized on bold font.

In the subgroup analysis, we also found that the incidence of PE in the LDA group was higher than that in the control group (57.14% vs. 33.33%) in women with chronic hypertension (*P* interaction = 0.0003). In addition, there were interactions between aspirin usage with age (≥ 35 or < 35 years), BMI (≥ 30 kg/m^2^ or < 30 kg/m^2^), history of PE, and family history of PE (Table 4).

**Table 4:** The incidence of PE between two groups according to maternal ACOG risk factors

Risk factor	Total	*Number of participants*		PE patients, n(%)		OR (95%CI)	*P*	*P* for interaction
		LDA	Control	LDA	Control			
Age (years)								**0.0023**
≥ 35	114	42	72	3 (7.14%)	10 (13.89%)	0.48 (0.12–1.84)	0.367	
< 35	152	64	55	9 (14.06%)	24 (43.64%)	**0.32 (0.14–0.75)**	**0.007**	
BMI(kg/m^2^)								**0.0331**
≥ 30	56	26	30	4 (15.38%)	9 (30.00%)	0.42 (0.11–1.59)	0.196	
< 30	210	89	121	8 (8.99%)	25 (20.66%)	**0.38 (0.16–0.89)**	**0.022**	
History of PE								**0.0002**
Yes	10	3	7	0 (0.00%)	5 (71.43%)	–	0.083	
No	256	112	144	12 (10.71%)	29 (20.14%)	**0.48 (0.23–0.98)**	**0.041**	
Family history of PE								**0.0013**
Yes	10	4	6	0 (0.00%)	4 (66.67%)	–	0.071	
No	256	111	145	12 (10.81%)	30 (20.69%)	**0.47 (0.23–0.96)**	**0.034**	
Pre-existing diabetes								0.0654
Yes	19	11	8	2 (18.18%)	2 (25.00%)	0.67 (0.07–6.11)	0.719	
No	247	104	143	10 (9.62%)	32 (22.38%)	**0.37 (0.17–0.79)**	**0.008**	
Chronic hypertension								**0.0003**
Yes	10	7	3	4 (57.14%)	1 (33.33%)	2.67 (0.16–45.13)	0.417	
No	256	108	148	8 (7.41%)	33 (22.30%)	**0.28 (0.12–0.63)**	**0.001**	
Nulliparous								0.0716
Yes	133	56	77	7 (12.50%)	17 (22.08%)	0.50 (0.19–1.31)	0.156	
No	133	59	74	5 (8.47%)	17 (22.97%)	**0.31 (0.11–0.90)**	**0.025**	
Multiple pregnancy								0.0626
Yes	57	24	33	3 (12.50%)	6 (18.18%)	0.64 (0.14–2.88)	0.720	
No	209	91	118	9 (9.89%)	28 (23.73%)	**0.35 (0.16–0.79)**	**0.009**	

*PE* preeclampsia, *ACOG* American college of obstetricians and gynecologists, *LDA* low-dose aspirin, *OR* (95%CI), odds ratio (95% confidence interval), *BMI* body mass index.

*P*-values <0.05 are emphasized in bold font.

## Discussion

Our study demonstrated that in pregnant women identified as high risk for PE, the administration of 75 mg aspirin per day, especially initiated before 16 gestational weeks, could reduce the incidence of PE without increasing the risk of adverse maternal and neonatal outcomes. PE usually originates from suboptimal trophoblastic invasion that ultimately causes excessive inflammatory response and endothelial dysfunction, increased platelet aggregation, and thrombotic due to placental infarcts [1, 40]. Accordingly, cyclooxygenase (COX) is activated and prostacyclin (PGI2) synthase is inhibited, resulting in a rapid and unfavorable elevation of the thromboxane A2 (TXA2)/PGI2 ratio [40]; thereafter, TXA2 increases platelet aggregation and causes vasoconstriction, while the vasodilatory effect of PGI2 cannot compensate [41]. LDA could selectively and irreversibly inactivate COX and reverse the TXA2/PGI2 imbalance, suppressing the production of prostaglandins and thromboxane as well as inhibiting inflammation and platelet aggregation, thus alleviating the syndromes or risk of PE [42]. Furthermore, Sibai et al. [43] noted that more than 90% of pregnancies had a significant decrease of TXA2 after taking 60–80 mg of aspirin per day for a few days, which also supported that 75 mg of aspirin may be a sufficient dosage for the prevention of PE.

A meta-analysis of 45 RCT studies found that LDA initiated before 16 gestational weeks [relative risk (RR) = 0.57, 95%CI = 0.43–0.75] was more likely to produce favorable preventive outcomes for PE compared to that after> 16 gestational weeks (RR = 0.81,95%CI = 0.66–0.99), which was in accordance with our finding [44]. In addition, Bujold et al. found that initiation of LDA started at 16 weeks of gestation or earlier was associated with a significant reduction in the incidence of PE (*RR* = 0.47, 95% CI = 0.34–0.65), whereas initiated after 16 weeks did not affect the risk of PE (*RR* = 0.81, 95% CI = 0.63–1.03) [45]. The results also concur with the recent review of Chaemsaithong et al. who reported that LDA should be started before 16 weeks of gestation, and PE screening should be done during the first trimester of pregnancy[46]. This may be because the trophoblast invasion of the uterine spiral arteries usually begins around 8 weeks and completes at approximately 16 weeks of gestation, and therefore prophylaxis for PE should start no later than 16 weeks of gestation [14].

A large RCT from China concluded that LDA prophylaxis may be ineffective for PE in patients with chronic hypertension, as the occurrence of PE was not statistically different between the LDA group (24.1%) and the control group (23%) [36]. In similar, the ASPRE trial found that among participants with chronic hypertension, the incidence of PE in the LDA (10.2%) was higher than that in the placebo (8.2%) groups [47]. Women with chronic hypertension often have symptoms of endothelial dysfunction and inflammation before pregnancy and may be more prone to placental damage than its counterparts [48, 49]. Our results were consistent with these findings, suggesting that the beneficial effect of LDA in the prevention of PE may be weakened in pregnancies with chronic hypertension. Hence, more RCT studies are needed to determine whether the preventive effect of LDA for PE is applicable to pregnant women with chronic hypertension.

## Strengths and limitations

The current study used real-world data, which is more easily generalizable to clinical usage. Admittedly, there were still some limitations in our study. Firstly, we failed to objectively evaluate aspirin compliance in terms of tablet intake, but only asked patients whether they were taking their medication as prescribed. Secondly, the initial time of aspirin administration in the current study was between 12 and 24 weeks of gestation, because pregnant women had a different time of their first prenatal appointment in real-world clinical practice. However, this also gave us the opportunity to reveal that LDA was more effective when administered before 16 weeks of gestation. Finally, the total sample size of the current study was relatively small, which may limit our power to perform some subgroup analyses. Therefore, additional real-world studies of larger samples are warranted.

## Conclusion

In summary, our study found that 75 mg per day of aspirin can reduce the incidence of PE in high-risk pregnancies and use LDA prior to 16 weeks of gestation is more effective in real-world clinical practice.

## Data Availability

The data sets generated and/or analyzed during the current study are not publicly available due to data protection regulations but are available from the corresponding author on reasonable request.

## Abbreviations

PE: Preeclampsia
CVD: Cardiovascular disease
ASPRE: Aspirin for evidence-based preeclampsia prevention
LDA: Low-dose aspirin
ACOG: American college of obstetricians and gynecologists
USPSTF: United States Preventive Services and Task Force
WHO: World Health Organization
SMFM: Society for Maternal–Fetal Medicine
RCT: Randomized controlled trial
BMI: Body mass index
SBP: Systolic blood pressure
DBP: Diastolic blood pressure
S/D: Systolic/diastolic
NICU: Neonatal intensive care unit
OR: Odds ratio
CI: Confidence intervals
SAS: Statistical analysis system
COX: Cyclooxygenase
TXA2: Thromboxane A2
PGI2: Prostacyclin
